# Osteolytic metastases from a pheochromocytoma presenting as multiple limb lameness and joint swelling in a dog: a case report

**DOI:** 10.3389/fvets.2025.1585969

**Published:** 2025-11-13

**Authors:** Madeleine Swindell, Timothy A. Bolton, Cassandra M. Powers, Ladislav Novotny, Jack Jarvis, Masahiro Murakami

**Affiliations:** 1 College of Veterinary Medicine, Purdue University, West Lafayette, IN, United States; 2 Department of Veterinary Clinical Sciences, College of Veterinary Medicine, Purdue University, West Lafayette, IN, United States; 3 Department of Comparative Pathobiology, College of Veterinary Medicine, Purdue University, West Lafayette, IN, United States; 4 California Animal Health and Food Safety Laboratory, University of California, Davis, Davis, CA, United States; 5 Department of Veterinary Sciences, Faculty of Agrobiology, Food and Natural Resources, Czech University of Life Sciences, Prague, Czech Republic; 6 Department of Pathobiology, School of Veterinary Medicine, St. George’s University, St. George’s, Grenada

**Keywords:** pheochromocytoma, joint swelling, adrenal neoplasia, metastasis, dog, lameness

## Abstract

Clinical signs due to distant bony metastasis from a malignant pheochromocytoma are rare in dogs, with the majority of reported cases presenting as single-limb lameness. This case report describes a 6-year-old neutered male mixed-breed dog presenting with multiple limb lameness and joint swelling that occurred over 3 weeks. Computed tomography revealed a mass in the right adrenal gland with extensive intrathoracic, intra-abdominal, and skeletal metastases. Because of the poor prognosis, the dog was humanely euthanized. A postmortem examination revealed a primary neoplasm of the right adrenal gland with metastases affecting the contralateral adrenal gland, kidneys, lungs, liver, and bones of the appendicular and axial skeletons. Immunohistochemistry confirmed a metastatic neuroendocrine carcinoma of the right adrenal gland, consistent with a pheochromocytoma. This case is a unique presentation of polyostotic metastases from a pheochromocytoma and emphasizes the importance of including this tumor in the differential diagnosis of dogs presenting with symptoms identical to those of polyarthritis.

## 1 Introduction

Pheochromocytomas are endocrine tumors originating from chromaffin cells in the adrenal medulla that secrete the catecholamines epinephrine and norepinephrine (1). Clinical signs are frequently the result of excessive catecholamine secretion and include lethargy, weakness, tachypnea, panting, and/or collapse; however, clinical signs related to a locally invasive, space-occupying mass and distant metastasis also occur, albeit less commonly (1–3). Because these clinical signs are variable and nonspecific, veterinarians may miss the presence of a pheochromocytoma, as evidenced by its frequent identification as an incidental finding (often at necropsy) in 50–60% of dogs (2, 3). An antemortem diagnosis of a pheochromocytoma is challenging and requires a high index of suspicion by the evaluating clinician.

While local invasion of nearby vasculature occurs in up to 50% of cases, distant metastasis is far less common (2, 3). Two retrospective studies (2, 3) found that less than 25% of dogs had distant metastasis; however, these studies and other isolated case reports have described widespread sites of metastasis, including the liver, lungs, kidneys, pancreas, prostate, spleen, heart, gastrointestinal tract, spinal canal, brain, bones (ribs, humeri, calvaria, cervical vertebrae, scapulae, femurs, and tibiae), and peritoneum (2–11). When present, clinical signs of distant metastasis primarily involve the musculoskeletal and nervous systems (2–11). Here, we describe the case of a dog with osseous metastases from a pheochromocytoma that presented with multiple limb lameness and swollen joints, a clinical presentation identical to that of polyarthritis.

## 2 Case description

A 6-year-old neutered male mixed-breed dog (weighing 24.3 kg) was evaluated for progressive, multiple limb lameness, joint swelling, and weight loss (4.1 kg) that occurred over 3 weeks.

### 2.1 Pertinent medical history

The dog was initially brought to the referring veterinarian for a weight-bearing right forelimb lameness and hyporexia (Day −21; Day 0 = referral date). The dog’s body temperature was normal (100.1 °F). Symptomatic treatment with capromorelin (3.2 mg/kg PO q24h) and carprofen (5.3 mg/kg PO q24h) was initiated; however, no improvement was observed. The dog re-presented to the same veterinarian 8 days later (Day −13) for intermittent, non-weight-bearing right forelimb lameness and persistent hyporexia. On physical examination, pain was not elicited on long bone palpation nor on joint flexion and extension, and no joint swelling was initially observed. The body temperature was slightly elevated (102.9 °F). Radiographs of the right humerus and antebrachium revealed no apparent bony abnormalities. Due to the concern for an infectious or inflammatory arthropathy, the dog was treated with prednisone (0.4 mg/kg PO q12h) and doxycycline (8.8 mg/kg PO q12h). Capromorelin was continued at the previously prescribed dosage, and the carprofen was discontinued.

Ten days later (Day −3), the dog was referred to an internist due to progressive lameness and swelling of the joints. On physical examination, the dog was still intermittently non-weight-bearing lame on the right forelimb and was now weight-bearing lame on the right hindlimb. Additionally, the right carpus, elbow, and stifle were swollen, and the dog had severe, diffuse muscle wasting, which was most prominent in the hind limbs and epaxial musculature. The dog had lost 4.1 kg since its initial presentation to the referring veterinarian almost 3 weeks prior. The body temperature was high-normal (102.5 °F).

Investigating the cause of the clinical signs included performing an abdominal ultrasound and arthrocentesis of the right carpus, elbow, and stifle. The abdominal ultrasound revealed a right adrenal mass invading the caudal vena cava. While awaiting the joint fluid cytology results, the dog was treated with amoxicillin-clavulanic acid (15.4 mg/kg PO q12h) and enrofloxacin (11.2 mg/kg PO q24h). Prednisone and capromorelin were continued at the previously prescribed dosages. Cytology of the joint fluid returned normal (<100 nucleated cells/μL; 85–90% mononuclear cells, 5–10% small lymphocytes, and <5% non-degenerate neutrophils) in all joints sampled.

### 2.2 Clinical presentation and investigation

Three days following discharge from the internist (day 0), the dog presented to the Purdue University Veterinary Teaching Hospital because it was unable to rise. On physical examination, the dog was recumbent and only able to stand with assistance. The right carpus, elbow, and stifle were swollen and painful upon palpation, and the previously noted muscle wasting was unchanged. The body temperature was high-normal (101.9 °F). A complete blood count and serum biochemistry profile revealed mild leukocytosis (19.1 × 10^3^/μL, reference interval (RI): 6.0–17.0 × 10^3^/μL) and increased alanine transaminase (ALT 341 IU/L, RI: 3–69 IU/L), alkaline phosphatase (ALP 1271 IU/L, RI: 20–157 IU/L), and γ-glutamyl transferase (GGT 46 IU/L, RI: 5–16 IU/L) activities. While these clinicopathologic alterations were most consistent with glucocorticoid administration, hepatic neoplasia (primary or metastatic) could not be excluded. A blood pressure measurement obtained via Doppler was 175 mmHg, which was suspected to be secondary to pain. A fentanyl constant rate infusion was initiated in an attempt to manage the pain; however, the dog remained painful as evidenced by vocalization following joint palpation and overall patient manipulation.

**Figure 1:**
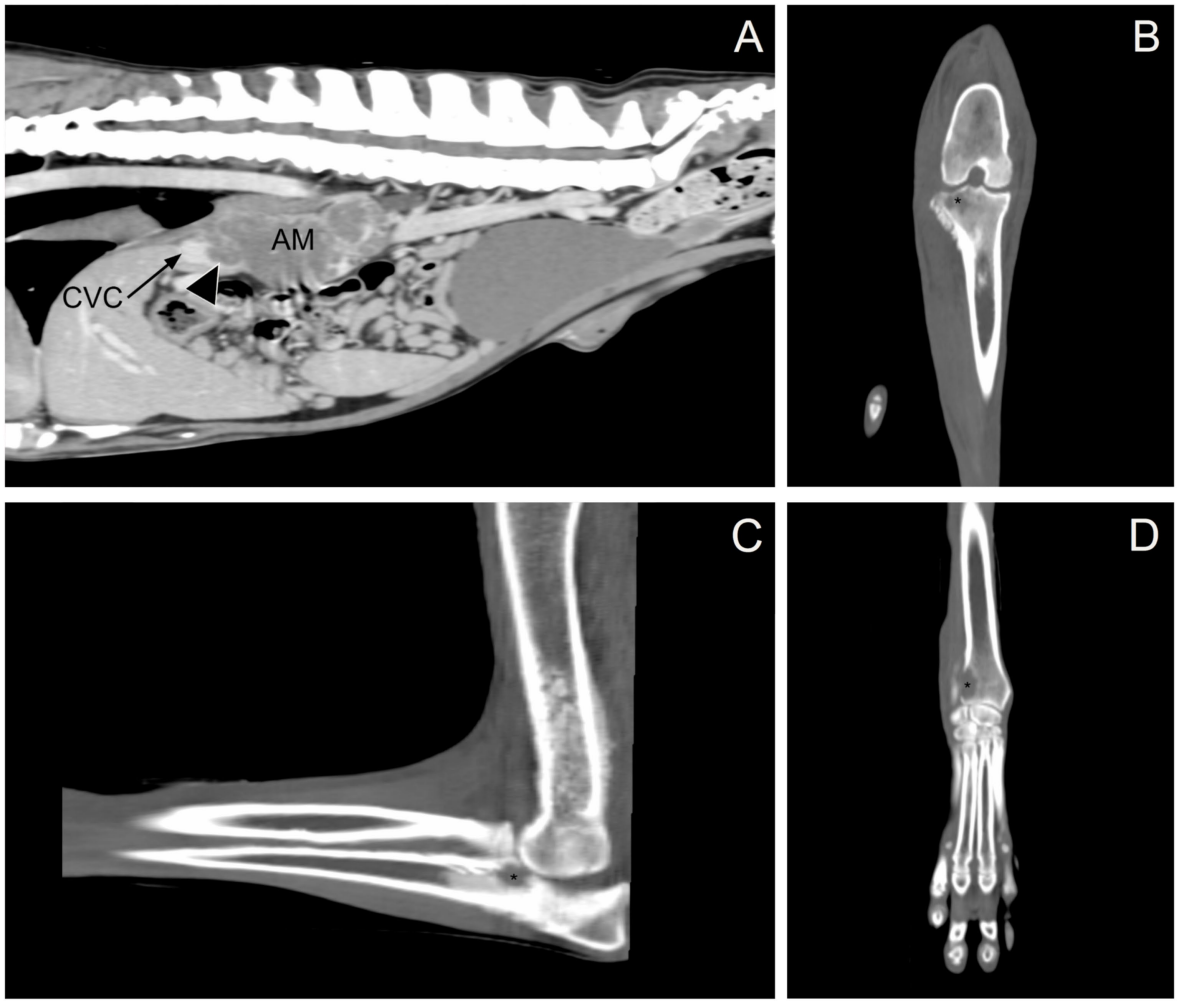
Computed tomography. **(A)** Sagittal, post-contrast image showing the right adrenal gland mass (AM) invading the caudal vena cava (CVC, black arrow; invasion, arrowhead). **(B)** Dorsal, pre-contrast image showing osteolysis in the proximal right tibia (black asterisk). **(C)** Sagittal, pre-contrast image showing osteolysis in the proximal right ulna (black asterisk). **(D)** Dorsal, pre-contrast image showing osteolysis in the distal right radius (black asterisk).

**Figure 2:**
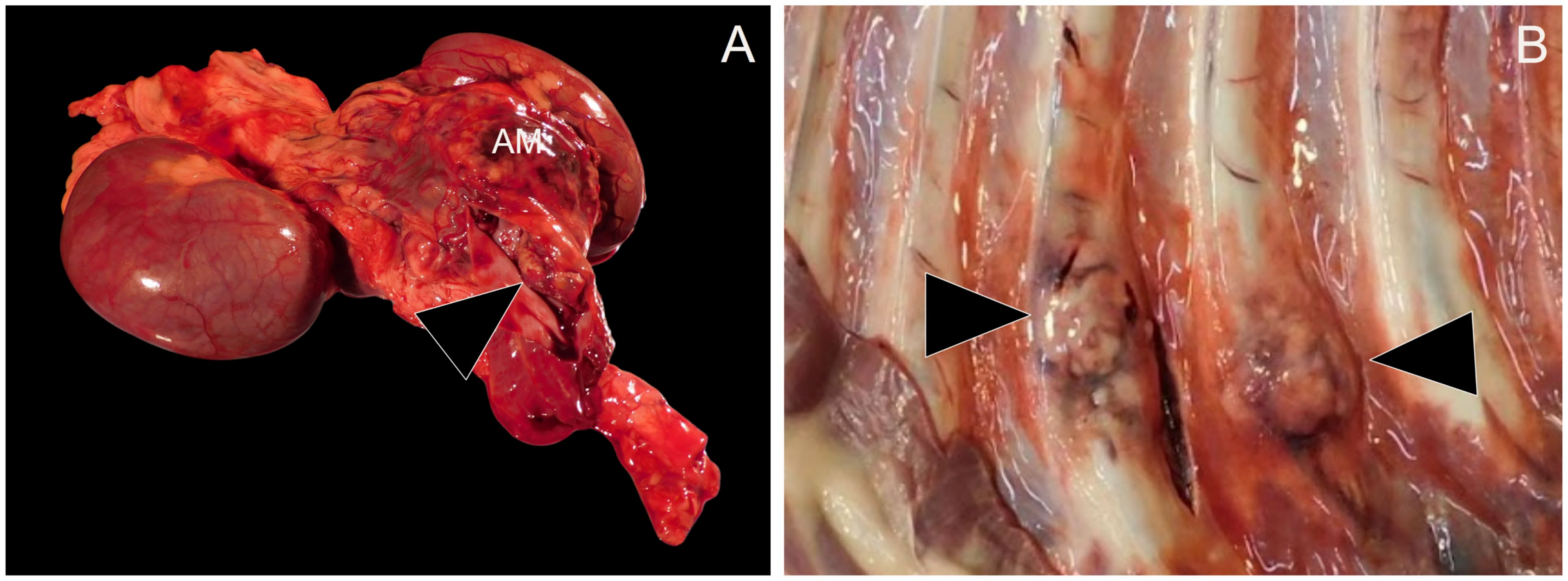
Necropsy examination. **(A)** Replacing the right adrenal gland is a 13 x 7 x 6 cm yellow-to-tan-to-red, firm, multinodular mass (AM). The adrenal mass extends into the lumen of the caudal vena cava (arrowhead). **(B)** Ranging in size from 1–3 cm in diameter at the level of the costochondral junction of the ribs are multiple firm to hard, mottled white to tan to red masses (arrowheads).

Given the previous finding of an adrenal mass in conjunction with joint swelling not attributable to inflammatory or infectious arthropathies, a CT scan of the thorax, abdomen, and thoracic and pelvic limbs was performed to investigate the local extent of the mass and for any metastatic disease. Imaging revealed the following abnormalities: (1) a large mass in the right adrenal gland extending into the caudal vena cava (Figure 1A); (2) nodular left adrenomegaly; (3) ill-defined, variably sized nodules and masses in multiple liver lobes; (4) ill-defined, variably sized nodules and masses in all lung lobes; (5) multifocal intra-abdominal lymphadenomegaly (medial iliac, internal iliac, splenic, hepatic, and lumbar aortic); and (6) multifocal regions of osteolysis involving the cervical, thoracic, lumbar, sacral, and caudal vertebrae, ribs, pelvis, right and left scapulae, right and left humeri, distal right and left radius, proximal right ulna, right radial carpal bone, distal right and left femur, and proximal right tibia (Figures 1B–D). Focal soft tissue thickening adjacent to the right carpus, elbow, and stifle was associated with osteolytic lesions in the distal right radius, right radial carpal bone, proximal right ulna, and proximal right tibia, resulting in periarticular swelling instead of true joint swelling (i.e., a true increase in synovial volume). Imaging findings supported primary malignant neoplasia of the right adrenal gland with local vascular invasion and widespread distant metastasis. Because of the extent of the disease in this dog and the associated poor prognosis, humane euthanasia was recommended.

During necropsy, gross examination revealed a 13 × 7 × 6 cm yellow-to-tan-to-red, firm, multinodular mass replacing the right adrenal gland. The mass extended into the adjacent caudal vena cava and occluded the lumen (Figure 2A). On cut section, the mass was diffusely mottled, ranging from tan to red to yellow. Multiple other neoplastic masses with a similar gross morphology were identified in the left adrenal gland, renal pelvis of the left kidney, caudate process of the caudate liver lobe, left lateral liver lobe, right cranial and caudal lung lobes, left caudal lung lobe, and the costochondral junctions of the right ribs 1, 4, and 7 and left ribs 4, 5, 7, and 8 (Figure 2B). Based on the gross examination at necropsy, the diagnosis was primary right adrenal gland neoplasia with disseminated metastases.

Tissues from the following organs were collected and processed for histopathological evaluation: heart, cerebrum, cerebellum, brainstem, lungs, liver, spleen, kidneys, thyroid glands, pancreas, adrenal glands, stomach, duodenum, jejunum, ileum, colon, urinary bladder, femoral bone marrow, right radial carpal bone, right distal radius, and ribs. All sampled tissues were fixed in 10% neutral buffered formalin, embedded in paraffin, sectioned at 4 μm, stained with hematoxylin and eosin, and reviewed by light microscopy.

**Figure 3:**
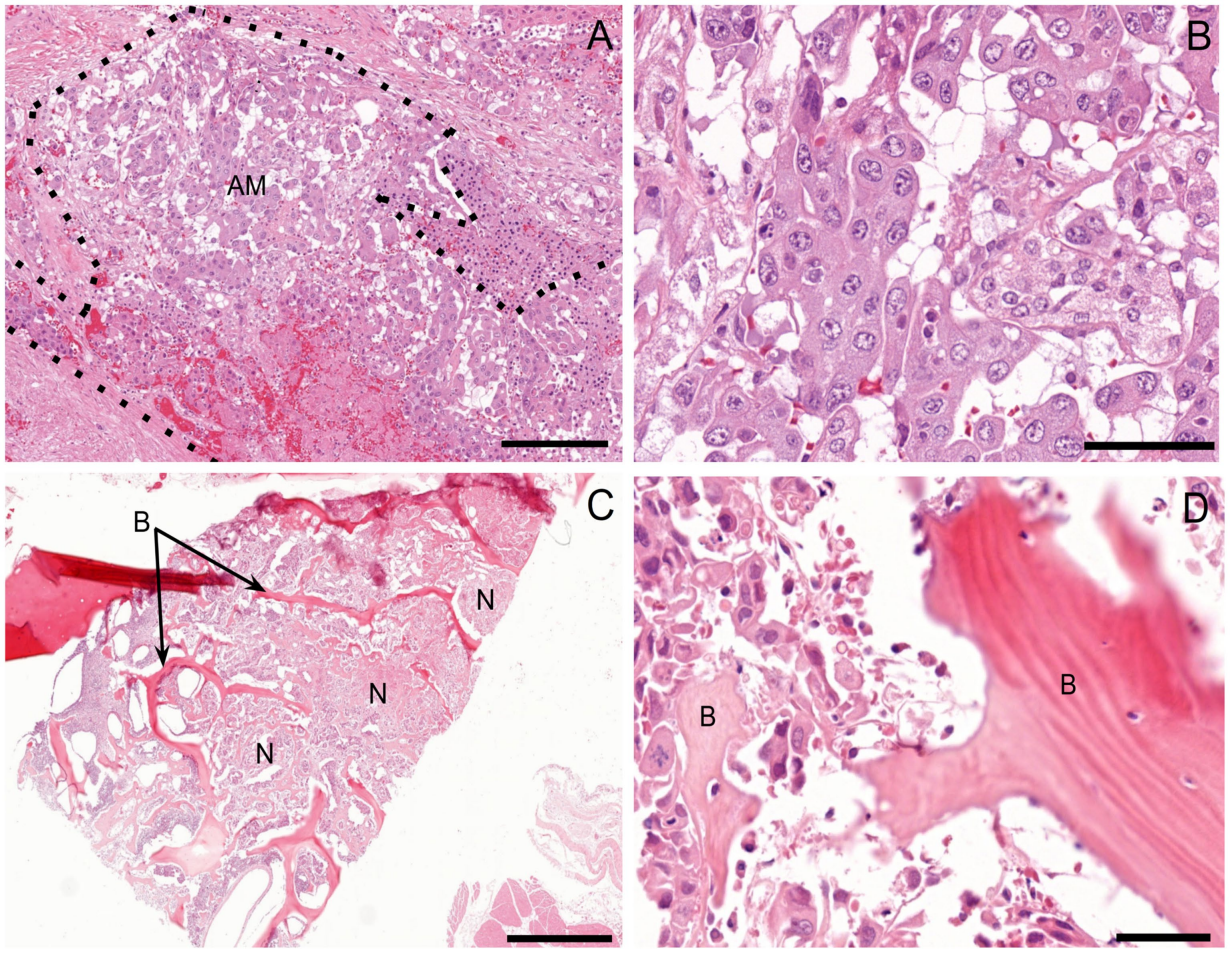
Histopathologic examination. **(A)** Right adrenal gland. Effacing the normal architecture of the adrenal gland is a nondemarcated and unencapsulated neoplasm (AM; outlined by dotted line). H&E 10x. Scale bar = 182 μm. **(B)** The neoplastic cells are polygonal to cuboidal and arranged in nests and packets, palisading along a delicate fibrovascular stroma. They have moderate amounts of eosinophilic, finely granular, and occasionally vacuolated cytoplasm, along with large, oval to irregular nuclei containing clumped chromatin and 1–3 small-to-moderate-size nucleoli. H&E 40x. Scale bar = 46 μm **(C)** Right radial carpal bone (B). Infiltrating, effacing, and surrounding variably sized islands of pre-existing woven bone are large islands and packets of a densely cellular, neoplasm (N). H&E 2x. Scale bar = 910 μm. **(D)** The neoplastic cells have a similar morphology to that of the right adrenal gland neoplasm and are interwoven with pre-existing bone (B). H&E 40x. Scale bar = 46 μm.

On histopathologic examination, an unencapsulated, poorly demarcated, densely cellular neoplasm was found within the right adrenal gland, effacing the normal architecture. This neoplasm was composed of polygonal to cuboidal cells supported by a fine fibrovascular stroma. The cells were arranged in clusters and packets, and occasional tubule-like structures, divided by moderate amounts of dense fibrocollagenous tissue (Figure 3A). The neoplastic cells had inconsistently distinct cell borders with moderate to large amounts of an eosinophilic, rarely vacuolated cytoplasm. Nuclei were round to irregular with stippled chromatin and up to three condensed nucleoli. Anisocytosis and anisokaryosis were moderate to marked (Figure 3B). There were 25 mitotic figures in 2.37 mm^2^ (equivalent to ten 400x high-power fields), including atypical mitotic figures. The majority of the neoplasm had been replaced by marked necrosis and hemorrhage. Multiple nodules and aggregates of neoplastic cells with similar microscopic morphology were identified within and effacing the normal parenchyma and architecture of the left adrenal gland, left kidney, right kidney, lung, liver, and ribs. Variably sized aggregates of neoplastic cells were also present in the medullary spaces of the femoral bone marrow, right radial carpal bone, and right distal radius (Figures 3C,D). Lymphovascular invasion was identified in multiple additional tissues, such as the urinary bladder wall. Based on the morphology of the neoplastic cells, presence of tubule-like structures, and lack of well-differentiated chromaffin cells, a carcinoma was initially suspected.

Immunohistochemistry (IHC) was pursued to further classify the tumor, with the relevant IHC procedures used listed in Table 1. Tyrosine hydroxylase, pancytokeratin, synaptophysin, chromogranin A, and thyroid transcription factor-1 (TTF-1) immunohistochemical stains were run in-house at the Purdue University Animal Disease Diagnostic Laboratory on the right adrenal gland, left adrenal gland, lung, liver, and bone tissues. On these same tissues, an α-fetoprotein (AFP) immunohistochemical stain was performed at the Cornell University Animal Health Diagnostic Center. Finally, on all aforementioned tissue aside from bone β-endorphin and metenkephalin immunohistochemical stains were performed at the Michigan State University Veterinary Diagnostic Laboratory.

**Table 1:** Antibodies used for immunohistochemistry.

Antigen	Vendor	Clone	Dilution	Incubation time	Temperature (°F)	Antigen retrieval	Detection method
Tyrosine Hydroxylase	Millipore	AB152	1:500	24–72 h	39.2	HIER-C	DAB
Pancytokeratin	Dako	MNF116	1:400	30–60 min	68–72	HIER-C	DAB
Synaptophysin	Neomarkers	SP11	1:200	30–60 min	68–72	HIER-C	DAB
Chromogranin A	Neomarkers	Not Available	1:100	30 min	68–72	HIER-C	DAB
TTF-1	Dako	8G7G3/1	1:400	30 min	68–72	HIER-C	DAB
ɑ-fetoprotein	Leica	C3	Not Available	30 min	68–72	HIER-C	DAB
β-endorphin	Dako	Not Available	1:50	12–24 h	39.2	HIER-C	DAB
Metenkephalin	Leica	Not Available	1:100	30–60 min	68–72	HIER-C	DAB

DAB, 3,3′-diaminobenzidine chromogen; HIER-C, heat-induced epitope retrieval-citrate buffer; TTF-1, thyroid transcription factor-1.

**Figure 4:**
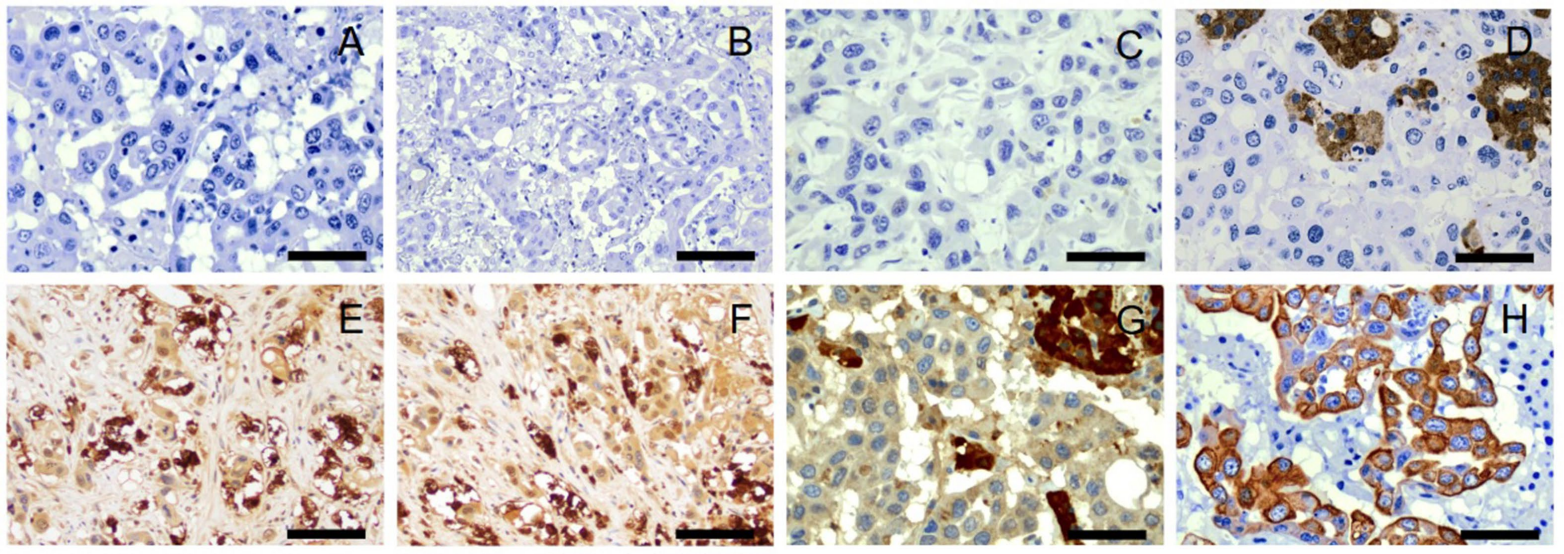
Immunohistochemistry, right adrenal gland. No immunolabeling for tyrosine hydroxylase **(A)**, thyroid transcription factor-1 **(B)**, *α*-fetoprotein **(C)**, synaptophysin **(D)**, *β*-endorphin **(E)**, or metenkephalin **(F)** is present in neoplastic cells. There is strong immunolabeling of normal background chromaffin cells for synaptophysin **(D)**. Tyrosine hydroxylase **(A)**, thyroid transcription factor-1 **(B)**, α-fetoprotein **(C)**, and synaptophysin **(D)**, 40x. Scale bars = 50 μm. β-endorphin **(E)** and metenkephalin **(F)**, 20x. Scale bars = 100 μm. Approximately 80–90% of neoplastic cells demonstrate weak to moderate cytoplasmic immunolabeling for chromogranin A **(G)** and strong cytoplasmic, submembranous immunolabeling for pancytokeratin **(H)**. There is strong immunolabeling of normal background chromaffin cells for chromogranin A **(G)**. Chromogranin A **(G)** and pancytokeratin **(H)**, 40x. Scale bars = 50 μm.

There was no immunolabeling for tyrosine hydroxylase, TTF-1, AFP, synaptophysin, β-endorphin, or metenkephalin within the neoplastic cells of the right adrenal gland (Figures 4A–F). Approximately 80–90% of the neoplastic cells in the right adrenal gland exhibited weak-to-moderate cytoplasmic immunolabeling for chromogranin A and strong cytoplasmic, mostly submembranous immunolabeling for pancytokeratin (Figures 4G,H). Similar immunolabeling results were found for all immunohistochemical stains in all other examined tissues. These results are summarized in Table 2 and confirm a diagnosis of metastatic neuroendocrine carcinoma of the right adrenal gland. A malignant pheochromocytoma with metastasis was the final diagnosis based on gross findings, microscopic findings, and IHC results.

**Table 2:** Summary of immunohistochemistry results.

Antigen	Right Adrenal Gland	Left Adrenal Gland	Lung	Liver	Bone
Tyrosine Hydroxylase	Negative	Negative	Negative	Negative	Negative
Pancytokeratin	Strong	Strong	Moderate to Strong	Moderate to Strong	Moderate to Strong
Synaptophysin	Negative	Negative	Negative	Negative	Negative
Chromogranin A	Weak to Moderate	Weak	Strong	Moderate to Strong	Moderate to Strong
TTF-1	Negative	Negative	Negative	Negative	Negative
*ɑ*-fetoprotein	Negative	Negative	Negative	Negative	Negative
*β*-endorphin	Negative	Negative	Negative	Negative	Not Available
Metenkephalin	Negative	Negative	Negative	Negative	Not Available

TTF-1, thyroid transcription factor-1.

## 3 Discussion

Clinical manifestations of distant metastatic disease in dogs with malignant pheochromocytomas are rarely reported in the veterinary literature, largely as isolated case reports (2–11). Among the reported cases of pheochromocytomas with osseous metastasis, the musculoskeletal and nervous systems are the most commonly affected, with clinical signs including epistaxis (2), pathologic fracture (4, 11), single-limb lameness (6, 7, 10), cervical pain (4), tetraparesis (8), paraparesis (8, 9), paraplegia (5), and seizures (3). In contrast to what has been observed in dogs, likely partially due to the paucity of reported cases, bone metastases occur in up to 70% of humans with malignant pheochromocytomas and are associated with at least one skeletal-related event subsequent to diagnosis. Despite being documented more frequently in humans, the metastatic behavior of this tumor appears similar in humans and dogs, as evidenced by a similar profile of (a manifestation of disease secondary to bone metastases) skeletal-related events in both species. These events include pain, pathological fracture, spinal cord compression, and hypercalcemia (12, 13). Not only does our case add to the repertoire of published veterinary cases documenting clinical signs related to distant bony metastases from a pheochromocytoma, but it also describes a novel clinical presentation of these metastases.

Paraneoplastic polyarthritis is an inflammatory joint disease that is associated with the presence of a solid tumor distant from the joints (14). While its pathogenesis is not well understood, multiple mechanisms, such as immune complex deposition in the synovium and molecular mimicry between tumor antigens and the synovium, have been postulated (14, 15). In humans, paraneoplastic polyarthritis has been reported in association with a metastatic pheochromocytoma; however, no such report exists in dogs (16). Because of the physical examination findings in our case, a diagnostic investigation for polyarthritis was justifiably pursued prior to the dog’s presentation at the Purdue University Veterinary Teaching Hospital; however, a neutrophilic inflammatory response in the synovial fluid was not present, which excluded a diagnosis of type IV (neoplasia-related) idiopathic polyarthritis and other types of immune-mediated polyarthritis (17, 18). The presence of a known adrenal mass highlighted the need to consider alternative mechanisms by which neoplasia can induce clinical signs and physical examination findings that mimic polyarthritis, as this can better inform the next steps in the diagnostic plan. In this case, cross-sectional imaging of the limbs was pursued, revealing periarticular soft tissue thickening closely associated with the osteolytic metastases from the adrenal gland neoplasm. These imaging changes corresponded to the swelling observed during the physical examination and provided an alternative mechanism for such findings. Polyostotic metastases from a pheochromocytoma should be included in the differential diagnosis for a dog with a clinical presentation consistent with polyarthritis but that is determined not to have polyarthritis based on arthrocentesis.

A carcinoma of the right adrenal gland was originally suspected because of the morphology of the neoplastic cells, presence of tubule-like structures, and lack of well-differentiated chromaffin cells on histopathology; however, positive immunolabeling for chromogranin A confirmed that the tumor arose from the adrenal medulla, consistent with a pheochromocytoma (2, 19). Although the tumor was unexpectedly negative for immunolabeling to tyrosine hydroxylase and synaptophysin, 25 and 15% of pheochromocytoma cases, respectively, can stain negative for these immunohistochemical markers (2, 20). Unfortunately, neoplastic cells in the tumor exhibited negative immunolabeling for β-endorphin and metenkephalin, which are opioid peptides expressed by chromaffin cells. While these results did not exclude a pheochromocytoma, the absence of staining for these two immunohistochemical markers suggested a nonfunctional tumor. A nonfunctional pheochromocytoma does not produce significant quantities of catecholamines or their metabolites and can therefore stain negative for opioid peptides. Negative immunolabeling for TTF-1 and AFP excluded concurrent carcinomas of pulmonary and hepatic origin, respectively.

Despite distant metastatic disease being present in fewer than 25% of dogs with pheochromocytomas (2, 3), the organs affected by tumor spread are extensive and include the liver, lungs, kidneys, pancreas, spleen, gastrointestinal tract, heart, peritoneum, brain, spinal canal, prostate, and bones (2–11). Many of these organs were found to have metastatic disease either on advanced imaging or necropsy. Additional metastatic sites included the urethra, pelvis, radius, ulna, lumbosacral vertebrae, and bone marrow. Documentation of these new metastatic sites improves our understanding of how pheochromocytomas behave and the clinical signs they can theoretically cause, such as stranguria and pollakiuria in the case of urethral metastasis. In human cases, metastasis occurs via hematogenous and lymphatic routes (21). The pheochromocytoma in this case extended into and occluded the caudal vena cava upon gross examination at necropsy, and various organs exhibited lymphovascular invasion on histopathology, suggesting that metastasis occurred via both routes in the observed dog.

In humans and dogs, adrenalectomy is the definitive and preferred treatment for pheochromocytoma; however, in cases with metastases, the tumor is considered malignant, and surgery is contraindicated. In these cases, medical treatment targeting the neoplastic cells or blocking the adrenergic response to circulating catecholamine excess may be considered. Hereditary germline mutations, specifically of the succinate dehydrogenase gene, are responsible for up to 50% of the metastatic pheochromocytomas in humans (22, 23). This mutation results in the activation of angiogenesis pathways through the overexpression of vascular endothelial growth factor receptors, suggesting that abnormally regulated angiogenesis plays a role in the pathogenesis of metastatic pheochromocytomas (24). Recently, mutations in the same gene were discovered in a small group of dogs, implying a common tumorigenic pathway between species (25). Inhibitors of vascular endothelial growth factor receptors, such as the tyrosine kinase inhibitors sunitinib, lenvatinib, and toceranib, have demonstrated clinical efficacy in both humans and dogs suffering from metastatic pheochromocytoma (26–32); however, prospective clinical trials in larger cohorts of each species are needed to elucidate the true extent to which these drugs are effective. Phenoxybenzamine, an ⍺-adrenergic receptor antagonist, can control clinical signs due to catecholamine excess; however, it does not decrease hormone secretion or tumor growth. The effect of this treatment on survival in dogs not undergoing surgery is unknown. In this case, due to the rapid progression of symptoms (<1 month in duration) and severe debilitation (pain unresponsive to injectable opioids and an inability to stand) caused by the metastatic disease, humane euthanasia was recommended, as no treatment was expected to improve the outcome or even quality of life. This case emphasizes the need for heightened awareness of the spectrum of clinical signs caused by a pheochromocytoma, especially those related to local tumor invasiveness and distant metastasis. Earlier detection of the disease may have provided some treatment options and the potential to gather longitudinal data about the response to treatment.

Clinical signs of catecholamine excess are nonspecific (e.g., anorexia, weight loss, and lethargy) or related to the cardiorespiratory (e.g., tachycardia, tachypnea/panting, collapse, and hemorrhage) or neuromuscular (e.g., weakness, pacing, and seizures) systems. In cases of suspected pheochromocytoma, normetanephrine (the metabolite of norepinephrine that is released into the bloodstream from the adrenal medulla) can be measured in the urine or plasma to attempt a noninvasive diagnosis. A plasma-free normetanephrine level >5.52 nmol/L or a urinary normetanephrine-to-creatinine ratio greater than 4 times the upper limit of the reference range increases the probability of a pheochromocytoma to nearly 100% (33, 34). However, the dog in this case did not undergo normetanephrine testing as it would not have changed the outcome, nor, based on the initial presentation, was a pheochromocytoma clinically suspected until after the diagnostic investigation was completed and the owner opted for humane euthanasia.

## 4 Conclusion

Diffuse bony metastases from a pheochromocytoma should be considered in the differential diagnosis for a dog presenting with multiple limb lameness and joint swelling. This clinical presentation resembles polyarthritis, so diagnostic investigation into other causes of these clinical signs should be pursued if joint cytology is not supportive. In cases similar to this with distant metastases, treatment options are limited, and the prognosis remains poor. Further studies are necessary to evaluate potential treatment options in a larger cohort of dogs with metastatic disease.

## Data Availability

The original contributions presented in the study are included in the article/supplementary material, further inquiries can be directed to the corresponding author.
